# Effects of Batri-7 on fallopian tube tissues in a rat model of salpingitis based on the toll-like receptor 4/myeloid differentiation factor 88/nuclear factor kappa B signalling pathway

**DOI:** 10.3389/fimmu.2026.1672173

**Published:** 2026-04-20

**Authors:** Rong Yu, Qiutong Wu, Yanhua Xu, Tianling Xiao, Sachurila, Hui Li, Genyu Li

**Affiliations:** 1 Integrated Traditional Chinese and Western Medicine Gynecology, Affiliated Hospital of Inner Mongolia Minzu University, Integrated Traditional Chinese and Western Medicine Gynecology, Tongliao, Inner Mongolia, China; 2 Pharmaceutical Clinical Trial Institution, Affiliated Hospital of Inner Mongolia Minzu University, Tongliao, Inner Mongolia, China; 3 Department of Gynecology, Baotou Central Hospital, Baotou, Inner Mongolia, China; 4 School of Clinical Medicine, Inner Mongolia Minzu University, Tongliao, China

**Keywords:** Batri-7, gavage, proinflammatory factor, toll-like receptor 4/myeloid differentiation factor 88/nuclear factor kappa B signalling pathway, tubal inflammatory infertility

## Abstract

**Objective:**

This study aimed to explore the underlying mechanism of Batri-7 in tubal inflammatory infertility model rats treatment in the toll-like receptor 4 (TLR4)/myeloid differentiation factor 88 (MyD88)/nuclear factor kappa B (NF-κB) pathway.

**Methods:**

Fifty-three specific-pathogen-free-grade female Sprague–Dawley rats were selected, of which 10 were randomly assigned to the blank control group (normal rats). The remaining 43 rats were used to establish a salpingitis model, induced by mixed bacteria, and were randomly divided into a model group, a positive control group, a low-dose experimental group and a high-dose experimental group. The rats in the blank and model groups were gavaged with 10 mL·kg^-1^·d^-1^ distilled water, a Jingangteng Capsules suspension (0.4 g·kg^-1^) was used for the positive control group and a high-dose (0.5g·kg^-1^) and low-dose (0.3g·kg^-1^) once a day was used for the treatment groups. The rats were sacrificed after 28 days, and the fallopian tube tissue was collected after blood collection from the abdominal aorta. The contents of interleukin-1 beta (IL-1β), interleukin-10 (IL-10), intercellular adhesion molecule-1 (ICAM-1) and tumour necrosis factor-alpha (TNF-α) in plasma were detected by enzyme-linked immunosorbent assay. Protein expression was evaluated using immunohistochemistry and western blot.

**Results:**

Compared with the model group, the high-dose experimental group, the low-dose experimental group and the positive control group could significantly reduce the expression levels of IL-1β, ICAM-1 and TNF-α in plasma, increase the expression level of IL-10 (*p* < 0.05) and significantly reduce the expression of TLR4 and MyD88 proteins in fallopian tube tissues (*p* < 0.05). The improvement in each index in the high-dose experimental group was significantly better than that in the low-dose experimental group and the positive control group (*p* < 0.05).

**Conclusion:**

Batri-7 can exert anti-inflammatory effects and treat salpingitis by regulating the TLR4/MyD8/NF-κB signalling pathway.

## 1 Introduction

Chronic salpingitis, a prevalent inflammatory disorder of the female reproductive system or pelvic inflammatory disease (PID), primarily manifests as persistent inflammation of the fallopian tubes (1). Its pathogenesis is frequently associated with lower genital tract infections, contiguous inflammation spread from adjacent organs or a history of intrauterine surgical procedures (2). Due to the insidious clinical manifestations in certain patients, the condition is frequently overlooked, giving rise to persistent unresolved inflammation that may induce tubal lumen stenosis, obstruction or extensive peritubal adhesions, thereby compromising fertility and quality of life (3). Studies have demonstrated that severe salpingitis can damage the mucosal epithelium, leading to fimbrial adhesions, distal obstruction and hydrosalpinx (structural alterations that impair ovum pickup and transport), thereby increasing the risks of chronic pelvic pain, infertility and ectopic pregnancy (4). Among the aetiologies of female infertility, salpingitis obstruction accounts for approximately 30%–35% of infertile cases, with its prevalence demonstrating a year-by-year upward trend (5). Current therapeutic strategies for tubal infertility, primarily laparoscopic surgery and antimicrobial agents are limited by postoperative fallopian tube re-adhesion and obstruction, giving rise to recurrent infertility. Long-term use of antimicrobial drugs may also entail side effects, such as drug resistance and the risks of medication abuse. Hence, there is an urgent need to investigate innovative approaches for restoring tubal function (6).

Mongolian gynaecological theory posits that salpingitis constitutes the central pathomechanism in PID, with therapeutic strategies primarily focused on anti-infective interventions (7). Pelvic inflammations induced by pathogens such as *Ureaplasma urealyticum* and *Chlamydia trachomatis* are among the most prevalent aetiologies of tubal infertility. Effective management of these infections is therefore critical for restoring tubal function and preventing infertility. Batri-7, a Mongolian medicine composed of seven herbal ingredients – Folium Aconiti Kusnezoffii, *Terminalia chebula* Retz., Oxytropidis Myriophyllae, *Rubia cordifolia* L., Commiphora Mukul and mercuric sulphide – exhibits cooling properties and exerts effects in resolving sticky evil, clearing heat and detoxifying/relieving epidemic toxins. It is used in the treatment of various diseases caused by epidemic heat, sticky evil-induced infections or sticky heat, leveraging its multi-component, multi-target and multi-pathway mechanism (8). In Mongolian medicine, ‘sticky disease’ (Zhanbing) denotes localised infections or acute conditions induced by pathogenic microorganisms (e.g. bacteria, viruses) invading the body through the oral–nasal passages or sweat glands. Clinically, Batri-7 has exhibited significant efficacy in managing diarrhoea, ulcerative colitis, chronic cervicitis and *Helicobacter pylori* infection. Preliminary studies by our research group have identified characteristic chromatographic spots of Moschus, *T. chebula* Retz. and *R. cordifolia* L. in Batri-7 (9). Additionally, research has shown that the toll-like receptor 4 (TLR4), myeloid differentiation factor 88 (MyD88) and nuclear factor kappa B (NF-κB) signalling pathways play crucial roles in immune responses, inflammation and apoptosis processes (10, 11).

Building upon our previous findings, the present study aims to investigate the effects of Batri-7 on salpingitis in rats through the TLR4/MyD88/NF-κB signalling pathway, thereby elucidating its therapeutic mechanism against salpingitis.

## 2 Materials and methods

### 2.1 Establishment of a chronic salpingitis rat model

Fifty-three specific-pathogen-free-grade female Sprague–Dawley rats, aged 8 to 10 weeks and weighing 200 ± 20 g, were acquired from Liaoning Changsheng Biotechnology Co., Ltd. (Animal production license number: SCXK[Liao] 2018-0001). The rats were housed in the Laboratory of Laboratory Animals, Institute of Mongolian Medicine Pharmacology, Affiliated Hospital of Inner Mongolia Minzu University (room temperature 22 °C–24 °C, relative humidity 55%–56%). Four rats were housed per cage under a controlled environment (22 °C, 40% relative humidity, 12 h/12 h light–dark cycle). The bedding was changed every 2 days, cages were cleaned and disinfected by dedicated personnel and the rats had free access to food and water. Drinking equipment was sterilised every 2 days. Ten rats were randomly assigned to the normal control group (untreated, not subjected to modelling), and the remaining 43 rats were used to establish a chronic salpingitis model induced by mixed bacteria, following the ‘mixed bacteria inoculation method’ described in previous reports (11). *Escherichia coli* (FSCC149006), *Staphylococcus aureus* (FSCC223005) and *β-haemolytic Streptococcus* (FSCC225002) were obtained from Beijing Dongfang Sairui Biotechnology Co., Ltd. (Beijing, China) and cultured in a certified microbiology laboratory. A mixed bacterial suspension (3 × 10^9^ CFU/mL) was prepared by combining *E. coli*, *S. aureus* and *β-haemolytic Streptococcus* in a 2:1:1 ratio and diluting with sterile injectable water. After anaesthesia, the lower abdomen was prepped and disinfected. A vertical incision approximately 0.8–1 cm long was made in the subcutaneous lower abdomen to open the abdominal cavity, exposing the Y-shaped uterus. A total of 0.1 mL of a 3 × 10^9^ CFU/mL mixed bacterial suspension was injected into each fallopian tube, 0.5 cm from the junction with the uterine horns on both sides. The abdominal incision was sutured, disinfected and wrapped with sterile dry gauze. On day 10 post-modelling, three rats were randomly selected for morphological assessment of the fallopian tubes – criteria included visible adhesion with surrounding tissues, oedema and vascular dilation and thickening. Haematoxylin and eosin (H&E) staining (using a non-toxic environmental solution, supplied by Keyida Instrument Commercial, Horqin District) was used to assess epithelial detachment, fibrosis of the lamina propria and reduction and atrophy of muscle fibres replaced by fibrous tissue, with evident enlarged interstitial spaces indicative of severe oedema, confirming successful model creation. This study was approved by the Medical Ethics Committee of the Affiliated Hospital of Inner Mongolia Minzu University (Ethics Approval No.: NM-LL 2025-01-17-17).

### 2.2 Model medication administration

Batri-7 (Approval No.: Nei Yao Zhi Bei Zi M20210233000) was prepared by the Mongolian Medicine Preparation Department of the Affiliated Hospital of Inner Mongolia Minzu University. Jingangteng Capsules (National Medical Products Administration Approval No. Z19991031) were produced by Hubei Furen Pharmaceutical Co., Ltd., China. Following the successful preparation of the model, the rats were divided into four groups, with 10 animals in each. The dosage for oral administration was calculated based on body surface area; the positive control group received an oral suspension of Jingangteng Capsules suspension (0.4 g·kg^-1^), the high-dose Batri-7 group received an oral suspension of Batri-7 (0.5 g·kg^-1^) and the low-dose Batri-7 group received an oral suspension of Batri-7 (0.3 g·kg^-1^). Both the model and blank group rats were gavaged with distilled water once daily for a continuous period of 28 days.

### 2.3 Enzyme-linked immunosorbent assay measurement of inflammatory markers

Enzyme-linked immunosorbent assay (ELISA) was utilised to determine the levels of interleukin-1 beta (IL-1β), interleukin-10 (IL-10), intercellular adhesion molecule-1 (ICAM-1) and tumour necrosis factor-alpha (TNF-α) in rat plasma. After the final drug administration, the rats were fasted for 24 h (water ad libitum) and anaesthetised via intraperitoneal injection of 3% pentobarbital sodium (30 mg.kg^-1^). Blood samples were collected from the abdominal aorta into anticoagulant tubes, left at room temperature until distinct blood layering was visible and then centrifuged (3,000 r/min for 15 min at a radius of 16 cm). The supernatant was harvested as plasma, and levels of IL-1β, IL-10, ICAM-1 and TNF-α were measured according to the kit’s instructions. The ELISA kits for IL-1β, IL-10, TNF-α and ICAM-1, provided by Tianjin Sikosai Biotechnology Co., Ltd., were used to measure absorbance at 450 nm with a microplate reader (BIOTEK, USA, Model ELX-800).

### 2.4 Immunohistochemistry

Immunohistochemistry (IHC) was performed to detect the expression of TLR4 and MyD88 proteins in the left fallopian tube tissues of the rats. The tissues were embedded in paraffin and sectioned using a microtome (Leica, Germany, model RM2235) to a 5 μm thickness. The sections were deparaffinised to water and treated with antigen retrieval solution. Endogenous peroxidase activity was blocked using hydrogen peroxide, following which primary antibodies diluted in phosphate-buffered saline (PBS) (1:200) were added to completely cover the tissues and incubated in a humid chamber overnight at 4 °C. Horseradish peroxidase (HRP)-conjugated secondary antibody (goat anti-rabbit IgG-HRP, Thermo Fisher, USA, catalogue #31460) was diluted 1:500 in PBS and added to the tissues, then incubated at 37 °C for 60 min. After washing in PBS for 5 min, the sections were developed using 3,3’-diaminobenzidine chromogen (Maixin New Century, China, catalogue DAB-1031) and counterstained with haematoxylin (Solarbio, China, H8070). The samples were sealed with neutral gum and examined under an optical microscope (Olympus, Japan, model BX53) for the positive expression rate of TLR4 and MyD88 messenger RNA proteins. Positive expression areas were scanned and scored using a microscope imaging system (Olympus, Japan, model DP73), followed by statistical analysis.

### 2.5 Western blotting

Western blotting was conducted to evaluate the expression levels of TLR4 and MyD88 proteins in the right fallopian tube tissues of the rats. Proteins were extracted using a total protein extraction kit (Wanleibio, China, catalogue WLA019). The cell lysis buffer was prepared and aliquoted with the addition of 1% phenylmethylsulfonyl fluoride. After adding the lysis buffer to the samples, they were subjected to an ice bath for 5 min and centrifuged at 12,000 rpm for 10 min at 4 °C. The supernatant served as the protein extract. Protein concentration was quantified using a bicinchoninic acid protein assay kit (Wanleibio, China, catalogue WLA004). Sodium dodecyl sulphate polyacrylamide gel electrophoresis gels were prepared using a rapid gel preparation kit (Wanleibio, China, catalogue WLA013) to form 5% stacking gel and 9%, 12% separating gels. Protein samples were diluted with 5x Loading Buffer (Wanleibio, China, catalogue WLA005) and PBS, boiled in a water bath for 5 min and prepared as loading samples. A volume of 20 μL of each sample was loaded into the gel wells, and electrophoresis was conducted at a constant voltage of 80 V for 2.5 h. The membranes were incubated with diluted primary antibodies (TLR4, WL00196 and MyD88, WL02494 from Wanleibio, China) overnight at 4 °C on a shaker. After washing with TBST, HRP-conjugated secondary antibodies (goat anti-rabbit IgG-HRP, Wanleibio, China, catalogue WLA023) were added, and the sample was incubated at room temperature for 1 h. The bands were visualised using an enhanced chemiluminescence detection system (Wanleibio, China, catalogue WLA006), and images were captured on a gel imager. Relative protein expression levels were quantitatively analysed by comparing the grayscale values of the target proteins with those of the internal control, β-actin (Wanleibio, China, catalogue WL01372).

### 2.6 Statistical methods

Data were organised using SPSS 25.0 (IBM, Armonk, NY, USA). Quantitative data are presented as mean ± standard deviation. For normally distributed data, comparisons among multiple groups were performed using analysis of variance after confirming homogeneity of variance, and pairwise comparisons were conducted using Tukey’s test. A *p*-value of <0.05 was considered a statistically significant difference.

## 3 Results

### 3.1 Successful establishment of the salpingitis model confirmed by haematoxylin and eosin staining

**Figure 1:**
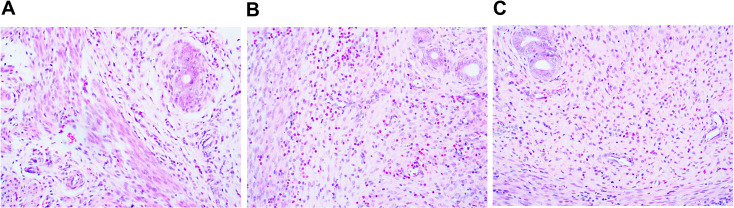
Histopathological evaluation of fallopian tubes in the model group **(A-C)** by H&E staining.

Following the establishment of the model, H&E staining was performed to examine the pathological features of murine fallopian tubes. Observations in the model group revealed infiltration of inflammatory cells within the lumen of the tubes, shedding of mucosal epithelium and localised oedema. Additionally, substantial infiltration of inflammatory cells was noted around the fallopian tubes, with some tissues appearing loosely structured and oedematous (**Figure 1**).

### 3.2 Batri-7 significantly modulates inflammatory cytokine expression in rat fallopian tube tissues

In comparison with the blank control group, the model group showed significant increases in IL-1β, ICAM-1 and TNF-α and a significant decrease in IL-10; these differences were statistically meaningful. Between the positive control group and the model group, there were notable reductions in IL-1β, ICAM-1 and TNF-α, with an increase in IL-10, all statistically significant. Comparisons between the experimental and model groups revealed significant decreases in IL-1β, ICAM-1 and TNF-α and an increase in IL-10, with all differences achieving statistical significance. The high-dose experimental group had significantly lower levels of IL-1β, ICAM-1 and TNF-α than both the low-dose experimental group and the positive control group, whereas IL-10 levels were significantly elevated, with statistical significance noted (*p* < 0.05, *p* < 0.01, Tukey’s test) (**Table 1**).

**Table 1:** Comparative levels of inflammatory cytokines in salpingitis tissues of rats ( $\bar{x}\pm s$).

Group	*n*	IL-1β	ICAM-1	TNF-α	IL-10
Blank group	10	80.44 ± 9.59	23.37 ± 4.08	137.36 ± 19.28	292.88 ± 21.73
Model group	10	212.08 ± 9.93^#^	75.13 ± 1.72^#^	450.71 ± 30.01^#^	90.74 ± 17.05^#^
Experimental advanced quantities	10	111.76 ± 11.69*	31.29 ± 4.12*	212.53 ± 30.69*	251.93 ± 23.08*
Experiment with low-level quantities	10	170.98 ± 15.38*	50.96 ± 3.47*	351.60 ± 24.80*	163.20 ± 18.43*
Positive control group	10	142.98 ± 13.57*	45.81 ± 5.16*	256.00 ± 16.63*	245.16 ± 20.94*

Comparisons among groups were performed by one-way ANOVA followed by Tukey’s *post hoc* test. # signifies a significant deviation compared to the blank group, and * indicates a significant deviation compared to the model group.

### 3.3 Batri-7 suppresses toll-like receptor 4 and myeloid differentiation factor 88 expression in rat salpingitis tissues as assessed by immunohistochemistry

**Figure 2:**
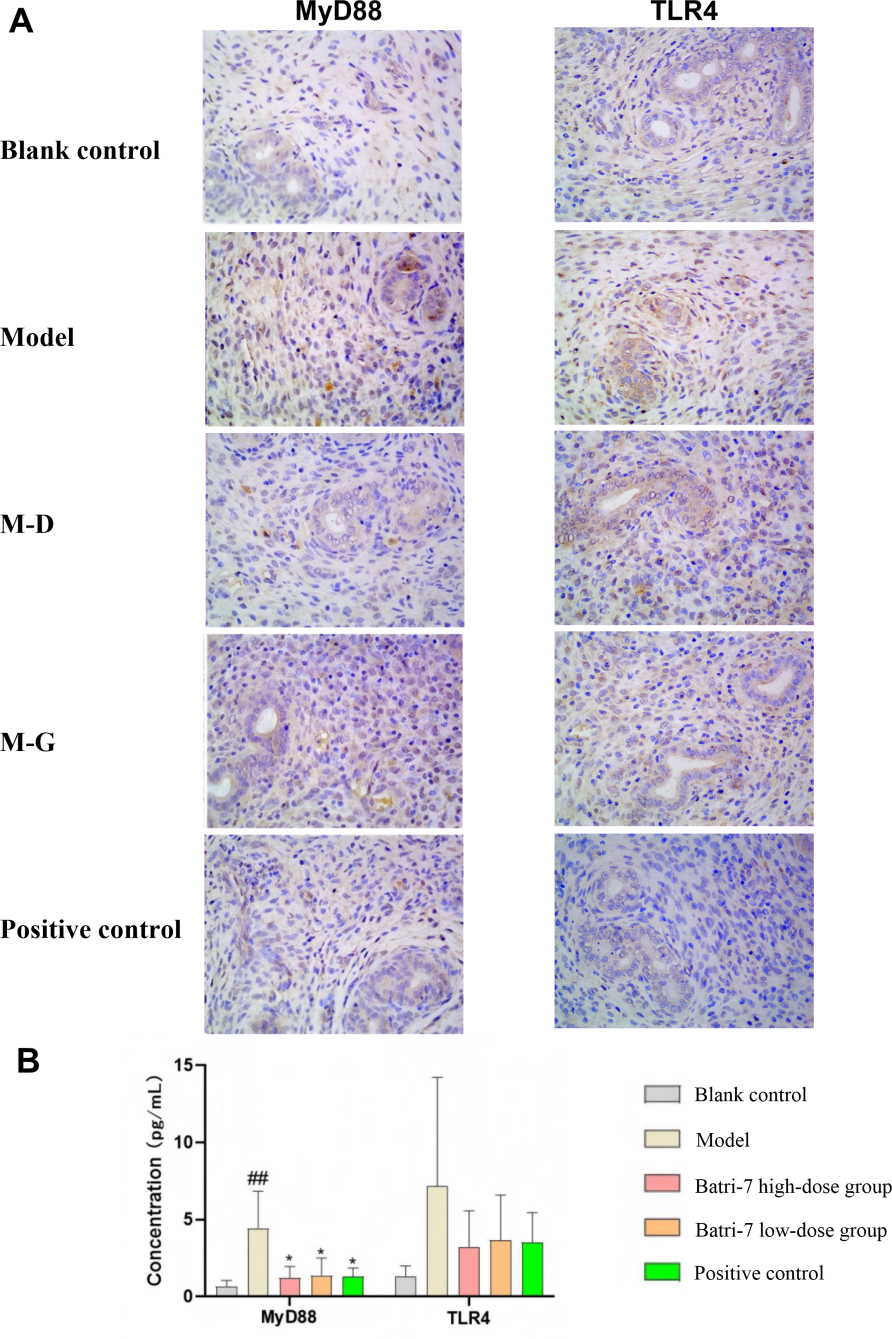
Immunohistochemical (IHC) detection of MyD88 and TLR4 protein expression. **(A)** IHC detection of MyD88 and TLR4 expression. **(B)** The statistical analysis of IHC results in **(A)** Statistical significance was determined by one-way ANOVA with Tukey’s multiple comparisons test. Compared to the blank control group, ^##^*P*<0.01; compared to the model group, ^*^P<0.05. M-D, Batri-7 low-dose group; M-G, Batri-7 high-dose group.

Immunohistochemistry was used to assess the expression of TLR4 and MyD88 proteins across different groups. The findings demonstrated that the expression level of MyD88 was significantly elevated in the model group compared with the blank control group. In contrast, both the positive control group and the experimental group exhibited significantly reduced MyD88 expression relative to the model group (all *p* < 0.05) (**Figure 2**).

### 3.4 Batri-7 dose-dependently inhibits toll-like receptor 4 and myeloid differentiation factor 88 protein expression as determined by western blot analysis

**Figure 3:**
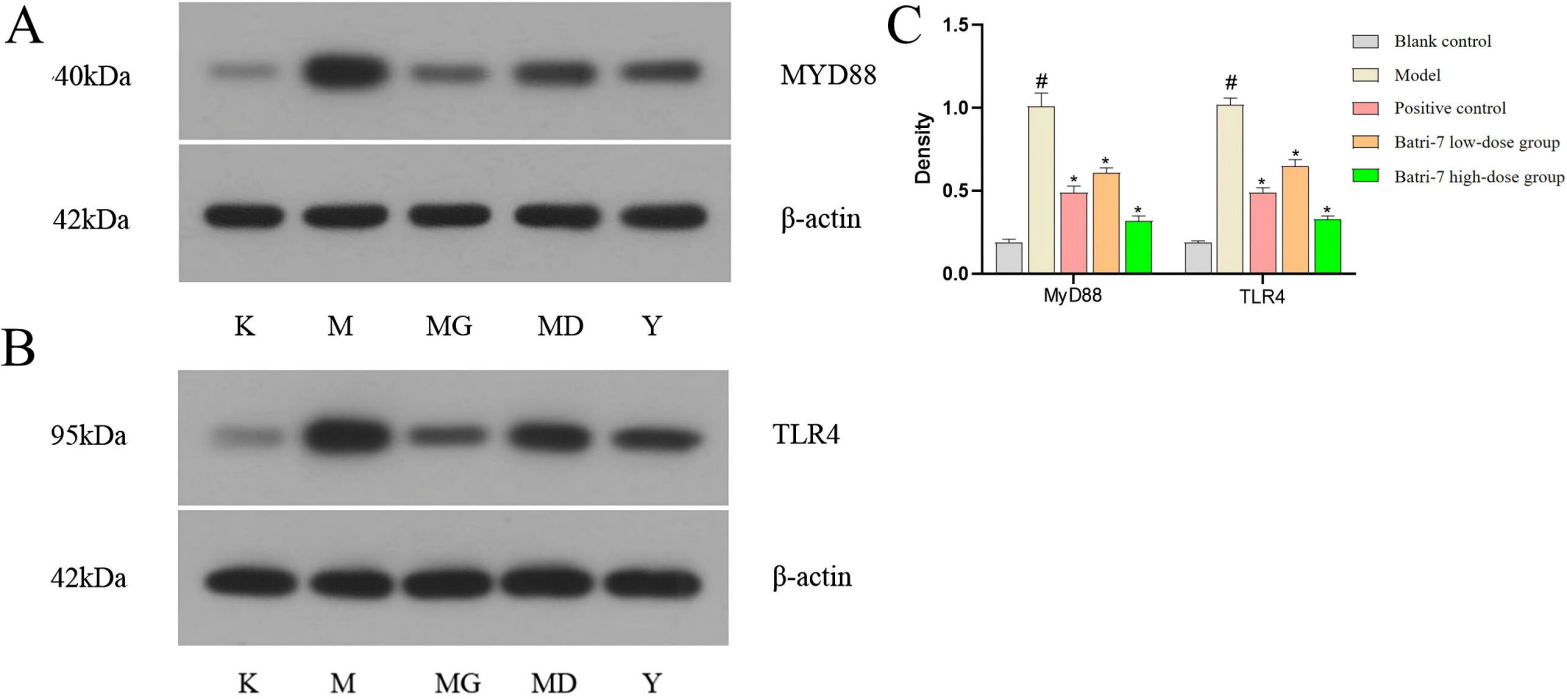
Western blot analysis for MyD88 and TLR4 protein expression. **(A)** Western Blot Analysis for MyD88 expression. **(B)** Western Blot Analysis for TLR4 expression. **(C)** The statistical analysis of Western Blot results in **(A, B)** Statistical significance was determined by one-way ANOVA with Tukey’s multiple comparisons test. Compared to the blank control group, ^#^*P*<0.05; compared to the model group, ^*^P<0.05. K, Blank control group; M, Model group; MG, Batri-7 high-dose group; MD, Batri-7 low-dose group; Y, Positive control group.

Western blot analysis indicated that Batri-7 effectively inhibited the expression of MyD88 and TLR4 across varying doses, with the inhibition being more pronounced in the high-dose group (**Figure 3**). This suggests that Batri-7 may exert its anti-inflammatory or therapeutic action by downregulating the expressions of MyD88 and TLR4 in a dose-dependent manner.

## 4 Discussion

Salpingitis, a major cause of female infertility, is driven by persistent inflammation that results in structural damage and functional impairment of the fallopian tubes. With the rising incidence of sexually transmitted diseases and an increase in the number of induced abortions in recent years, the rate of infertility attributable to this disease has also increased considerably (12). Numerous studies highlight the pivotal role of the TLR4/MyD88/NF-κB signalling pathway in inflammatory responses triggered by various infectious diseases (10, 13). Pro-inflammatory cytokines IL-1β and TNF-α, together with the adhesion molecule ICAM-1, are key mediators of inflammatory responses, and their expression is largely regulated by the TLR4/MyD88/NF-κB signalling pathway. Activation of this pathway promotes NF-κB–mediated transcription of inflammation-related genes, leading to upregulation of IL-1β, TNF-α and ICAM-1, thereby amplifying inflammatory responses and facilitating inflammatory cell infiltration. In contrast, the anti-inflammatory cytokine IL-10 suppresses NF-κB activity and negatively regulates the production of these pro-inflammatory mediators. Therefore, inhibition of the TLR4/MyD88/NF-κB pathway contributes to attenuation of pro-inflammatory responses and restoration of immune homeostasis within the inflammatory microenvironment (13). Our findings reveal that the traditional Mongolian medicine compound Batri-7 significantly mitigates inflammatory damage in rat models of salpingitis-induced infertility by modulating this signalling pathway, underscoring its potential therapeutic mechanisms.

We established a rat model of salpingitis through intrauterine inoculation with mixed bacteria. Jingangteng Capsules are a traditional Chinese patent medicine prepared from the single herb Jingangteng and are widely used in clinical practice for the treatment of PID, adnexitis and salpingitis. Compared with other traditional Chinese patent medicines commonly used for these conditions, Jingangteng Capsules demonstrate superior efficacy in promoting blood circulation and resolving blood stasis. Therefore, Jingangteng Capsules were selected as an additional positive control in this study (14). Significant physiological changes in the fallopian tubes of the model group rats were observed, including mucosal epithelial shedding, inflammatory cell infiltration and tissue oedema, indicative of chronic inflammation onset. The ELISA results showed elevated levels of pro-inflammatory cytokines IL-1β, TNF-α and ICAM-1 in the plasma of these rats, whereas the anti-inflammatory cytokine IL-10 was notably decreased. Consistent with previous research, these outcomes suggest that hyperactivation of inflammatory pathways can cause damage to the fallopian tubes and impair fertility (15). Analyses post-treatment with Batri-7 showed that both high and low doses significantly reduced levels of pro-inflammatory cytokines and increased expression of IL-10. Western blot and IHC results demonstrated that Batri-7 significantly downregulated the expression of TLR4 and MyD88 proteins in fallopian tube tissues, moreso in the high-dose group, indicating a dose-dependent therapeutic effect. Earlier research confirmed that Batri-7 downregulates NF-κB p65, alleviating salpingitis in rats (16). These findings indicate that Batri-7 can modulate the TLR4/MyD88/NF-κB signalling pathway, thereby attenuating inflammatory responses in the fallopian tube wall. The pathogenesis of salpingitis-induced infertility is complex and typically associated with infections, intraluminal haemorrhage, inflammatory responses, ascending infections (such as endometritis and cervicitis) and sexually transmitted infections (such as non-gonococcal urethritis and gonorrhoea). These conditions can trigger local inflammation of the fallopian tube and endometrium, leading to vasodilation, increased permeability and excessive immune cell (e.g. leukocyte) infiltration (17). Pharmacological studies have shown that components in the Batri-7 formulation, such as kusnezoff monkshood root in combination with aloeswood, exhibit analgesic and mucolytic effects; *Astragalus mongholicus* shows mucolytic properties; musk facilitates opening orifices and promotes mucolysis; and madder root acts as an antidiarrhoeal and clears blood heat (18). Previous pharmacological studies have shown that several components of Batri-7 possess anti-inflammatory, antimicrobial and immunomodulatory properties, which may indirectly influence TLR4 activation by reducing pathogen-associated molecular patterns, such as bacterial components, or by modulating upstream inflammatory microenvironments. Based on existing evidence, we hypothesise that Batri-7 may interfere with the TLR4/MyD88 pathway by attenuating ligand-receptor activation, suppressing MyD88 recruitment or limiting downstream signal amplification, thereby reducing NF-κB–mediated transcription of pro-inflammatory cytokines. However, these mechanisms remain speculative and require further experimental validation. In models of salpingitis, the inhibition of the TLR4/MyD88/NF-κB pathway suggests that Batri-7 may exert anti-inflammatory effects by suppressing the activation of this inflammatory pathway and reducing the release of downstream inflammatory mediators. This action helps maintain the structural integrity and normal function of the fallopian tubes, ultimately improving the reproductive outcomes of the treated animals (19). Compared with the positive control group (Jingangteng Capsules suspension), the high-dose Batri-7 group exhibited superior efficacy in regulating inflammatory cytokines and signalling molecules, indicating greater potential in treating salpingitis-induced infertility.

Nonetheless, the study is not without limitations. The specific active components within Batri-7 that contribute to its anti-inflammatory effects are yet to be identified and warrant further isolation and characterisation. Additionally, long-term follow-up and clinical trials are essential to validate its safety and efficacy in humans. Collectively, our findings demonstrate that Batri-7 exerts protective effects against salpingitis-induced infertility through the inhibition of the TLR4/MyD88/NF-κB-mediated inflammatory response. This provides a scientific rationale for the application of Batri-7 in treating salpingitis-related conditions and underscores the significant value of traditional Mongolian medicine in the prevention and treatment of gynaecological inflammatory diseases.

## 5 Conclusion

This study demonstrates that Batri-7 effectively alleviates salpingitis-induced infertility in rats by inhibiting the TLR4/MyD88/NF-κB signalling pathway. Batri-7 significantly reduces pro-inflammatory cytokines (IL-1β, ICAM-1, TNF-α) and increases anti-inflammatory cytokine IL-10 levels, while downregulating TLR4 and MyD88 protein expression in fallopian tube tissues. These findings suggest Batri-7’s potential as a therapeutic agent for salpingitis-related infertility. However, further research is needed to identify its active components and validate its clinical efficacy and safety.

## Data Availability

The original contributions presented in the study are included in the article/supplementary material. Further inquiries can be directed to the corresponding author.
